# High-Resolution Replication Profiles Define the Stochastic Nature of Genome Replication Initiation and Termination

**DOI:** 10.1016/j.celrep.2013.10.014

**Published:** 2013-11-07

**Authors:** Michelle Hawkins, Renata Retkute, Carolin A. Müller, Nazan Saner, Tomoyuki U. Tanaka, Alessandro P.S. de Moura, Conrad A. Nieduszynski

**Affiliations:** 1 Centre for Genetics and Genomics, School of Life Sciences, Queen’s Medical Centre, The University of Nottingham, Nottingham NG7 2UH, UK; 2 Centre for Gene Regulation and Expression, The University of Dundee, Dow Street, Dundee DD1 5EH, UK; 3 Institute for Complex Systems and Mathematical Biology, SUPA, King’s College, The University of Aberdeen, Aberdeen AB24 3UE, UK

## Abstract

Eukaryotic genome replication is stochastic, and each cell uses a different cohort of replication origins. We demonstrate that interpreting high-resolution *Saccharomyces cerevisiae* genome replication data with a mathematical model allows quantification of the stochastic nature of genome replication, including the efficiency of each origin and the distribution of termination events. Single-cell measurements support the inferred values for stochastic origin activation time. A strain, in which three origins were inactivated, confirmed that the distribution of termination events is primarily dictated by the stochastic activation time of origins. Cell-to-cell variability in origin activity ensures that termination events are widely distributed across virtually the whole genome. We propose that the heterogeneity in origin usage contributes to genome stability by limiting potentially deleterious events from accumulating at particular loci.

## Highlights

• Deep sequencing reveals a high-resolution view of genome replication dynamics
• Genome-wide modeling and single-cell imaging reveal stochastic origin activity
• Origin activity determines the location of replication termination events
• Termination events are widely distributed across the whole genome

## Graphical Abstract

**Figure:**
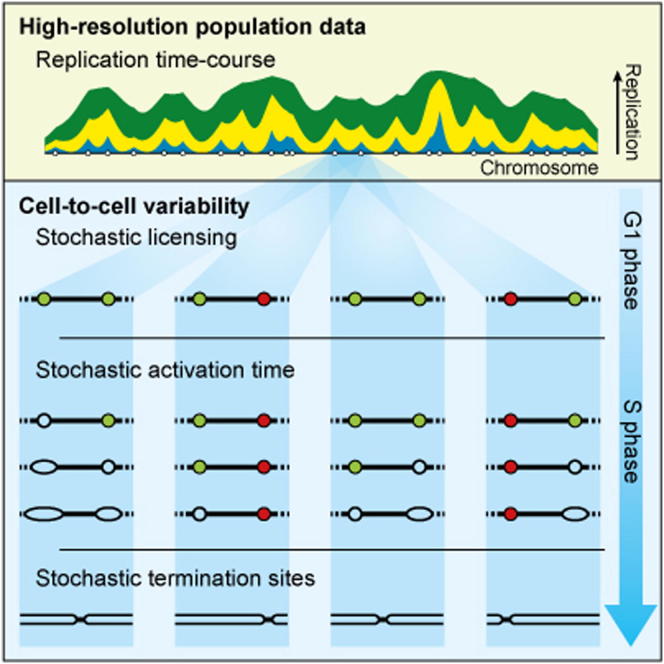

## Teaser

Little is known about what determines the genomic location of DNA replication termination sites. In this study, Nieduszynski and colleagues show that genome replication is highly variable between cells within a population. This variability arises from stochastic origin activity and results in replication termination sites being widely distributed across virtually the whole genome. The variability between cells in genome replication may contribute to genome stability by limiting potentially deleterious events from accumulating at specific loci.

## Introduction

DNA replication initiates at sites called origins of replication and must be completed before cell division. Eukaryotes depend on multiple origins to complete replication of their large linear chromosomes in a timely manner (Méchali, 2010; Siow et al., 2012). Origins are licensed to make them competent to activate by the assembly of a prereplication complex during late mitosis and G1 phase. During the subsequent S phase, a subset of these licensed origins initiates replication (Labib, 2010). Remaining licensed origins, called *dormant origins*, serve as backups, available to complete replication if required (Blow et al., 2011; Newman et al., 2013).

Eukaryotes have a distinct spatial and temporal pattern of genome replication that is dictated by the distribution and activity of replication origins. High-throughput experiments have enabled genome-wide characterization of the temporal order of replication (Gilbert, 2010). However, these ensemble approaches mask heterogeneity—that is each cell within a population is replicated from a different cohort of origins (Bechhoefer and Rhind, 2012). The heterogeneity is a consequence of individual origins being active in <100% of cell cycles; this is termed origin efficiency. Variable origin efficiency is a hallmark of eukaryotic genome replication and has been observed by a number of techniques (Friedman et al., 1997; Tuduri et al., 2010; Yamashita et al., 1997). The failure of an origin to activate can be due to the origin not being licensed or to being passively replicated, the latter of which gives rise to dormant origins. Passive replication can be a consequence of variation in origin activation time due to the stochastic nature of the molecular processes involved (de Moura et al., 2010; Raghuraman and Brewer, 2010). Currently, single-cell and single-molecule studies are not capable of measuring the kinetics of whole-genome replication. Although ensemble measurements mask stochastic origin activity (de Moura et al., 2010), in theory, a signature remains within the data allowing it to be quantified (Retkute et al., 2011).

Eukaryotic origin activation and replication dynamics have been extensively studied, but the nature of replication termination has not been widely explored. A minority of termination events are programmed by polar replication fork barriers (RFB). The RFB within the ribosomal DNA repeats are a conserved feature of eukaryotic replication and have been described in metazoa and yeasts (Dalgaard et al., 2009). However, most termination events occur when forks traveling in opposite directions collide. Termination sites are locally the latest replicating regions, but the stochastic nature of replication initiation makes it difficult to locate termination sites from ensemble replication timing data (de Moura et al., 2010). Fachinetti et al. (2010) tracked replication fork progression in *S. cerevisiae* to identify 71 termination zones (TER sites). To date, the proportion of termination events that takes place within these TER sites and the genome-wide distribution of replication termination have not been determined.

Here, we present the dynamics of genome replication in *S. cerevisiae* at high spatial and temporal resolution. We show that mathematical analysis of ensemble time-course data allows estimation of the stochastic properties of genome replication, such as the efficiency of each origin and the distribution of replication termination events. Independent experimental data, including single-cell measurements, support the inferred stochastic properties. Replication termination events are found to be widely distributed over virtually the whole genome. Previously reported TER sites correspond to maxima in the estimated distribution but to a minority of total termination events. A strain, in which three origins were inactivated, confirms that the location of termination events is primarily dictated by stochastic origin activation time. In summary, this study demonstrates that high-resolution ensemble methods can be used to quantify the heterogeneity of cellular processes.

## Results

### High-Resolution *S. cerevisiae* Replication Profiles

**Figure 1:**
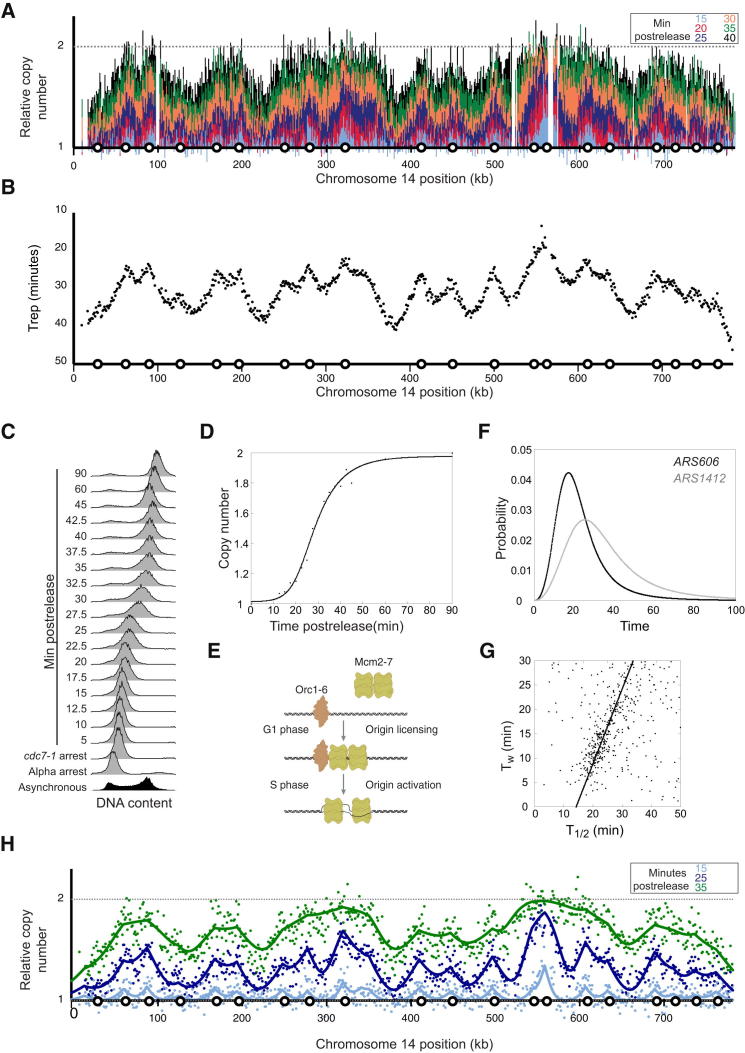
A High Spatial and Temporal Resolution View of Genome Replication (A) Replication profiles for chromosome 14. The six S phase time points (15, 20, 25, 30, 35, and 40 min) are relative to release from the *cdc7-1* zero time point. Each data point represents the extent of DNA replication in a 1 kb window. Circles on the x axis represent the location of replication origins. (B) Median replication time, Trep, derived from the time-course data shown in (A). (C) Flow cytometry data for the time-course experiment (see also Figure S1). (D) Quantification of the flow cytometry data that were used to normalize the deep sequencing data presented in (A). (E) The two temporally separate stages of replication origin function, licensing and activation, that the mathematical model captures. (F) Representative origin activation probability curves for an early activating origin (*ARS606*) and a late activating origin (*ARS1412*; see also Table S1). (G) Median origin activation time correlates with the width of the activation distribution (see also Figure S2). (H) Model fit (continuous line) to experimental replication-time-course data (points) for three representative time points.

We measured the replication dynamics of the *S. cerevisiae* genome (Figures 1A and 1B). Deep sequencing was used to measure the DNA copy number change, at 1 kb resolution, as each region of the genome was replicated. A highly synchronous S phase was achieved using a double arrest and release protocol (Figure 1C). Samples for deep sequencing were taken at 5 min intervals during S phase, with time points selected to maximize the information about S phase dynamics. Two nonreplicating samples (alpha factor arrest and *cdc7-1* arrest) were also sequenced to serve as controls. The extent of DNA replication at each time point was determined by comparison to the nonreplicating control samples.

It has been previously reported that active early origins can replicate during a *cdc7* arrest (Donaldson et al., 1998; Reynolds et al., 1989). This is known as “*escape*” replication and can be explained by residual Cdc7 activity at the nonpermissive temperature. To avoid escape replication, we optimized the *cdc7-1* arrest temperature and performed the arrest at the highest temperature from which the majority of cells released synchronously (Figure S1). Comparison of nonreplicating samples (*cdc7-1* arrest normalized to the alpha factor arrest) showed no evidence of escape replication. Therefore, each of the S phase samples was normalized to the *cdc7-1* nonreplicating control. Flow cytometry data were used to measure the change in copy number over time (Figures 1C and 1D) and to normalize the deep sequencing data (Yabuki et al., 2002).

The DNA copy number at each S phase time point allowed the extrapolation of the time at which each chromosomal coordinate had been replicated in half of the cells, Trep (Figure 1B). Previously, we have shown that sharp peaks in profiles are a consequence of defined replication origin positions. In contrast, smooth valleys result from the range of termination sites that are a consequence of stochastic origin activation time (de Moura et al., 2010; Retkute et al., 2011). However, the resolution of previous studies required smoothing that obscures the sharp origin peaks and could have contributed to the smooth termination valleys (de Moura et al., 2010). The high spatial resolution of the deep sequencing data negated the need for smoothing. Therefore, the sharp peak signatures of discrete origins can be resolved in the Trep data. The Trep profiles also showed smooth valleys, consistent with stochastic origin activation time and dispersed termination sites. Consequently, this ensemble data may allow quantification of the stochastic properties of genome replication.

### Quantitative Interpretation of Replication-Time-Course Data

An established model (de Moura et al., 2010; Retkute et al., 2011, 2012) was used to quantitatively interpret the genome-wide replication data and determine the stochastic properties. The model incorporates the temporally separated stochastic steps of origin licensing and activation (Figure 1E). Each origin is described by four properties: position, competence (p), median activation time (T_1/2_), and the width of the activation distribution (T_w_). Here, competence refers to the proportion of cells in which an origin is licensed. The stochastic activity of an origin is described by an activation curve (Figure 1F).

Using previously determined replication origin locations (Siow et al., 2012), the remaining origin properties could be derived by fitting the model to the experimental replication profiles (Figure 1H; Table S1). In this analysis, the average replication fork velocity was assumed to be constant and estimated to be 1.6 kb/min—comparable to independent estimates (de Moura et al., 2010; Sekedat et al., 2010; Tuduri et al., 2010; Yang et al., 2010). Analyses of the inferred origin properties showed that there is a positive correlation between T_1/2_ and T_w_ (Figure 1G); i.e., the later an origin activates, the more stochastic the activation time (Yang et al., 2010). No significant correlations were observed between other combinations of origin properties (Figure S2). Therefore, combining a mathematical model with high-resolution replication-time-course data allowed the estimation of stochastic origin properties.

### Stochastic DNA Replication Origin Activity

**Figure 2:**
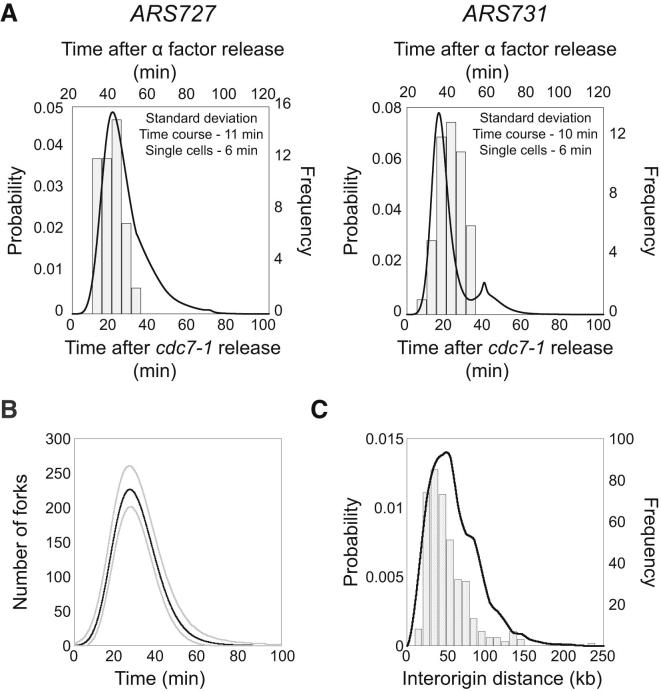
Origin Replication Time Is Stochastic (A) Replication time of *ARS727* (left) and *ARS731* (right) are shown as inferred from time-course data (continuous line; time relative to *cdc7-1* release shown on lower x axis) and single-cell measurements (histogram; time relative to α factor release shown on upper x axis). Equivalent S phase time points for *cdc7-1* and α factor release were determined by flow cytometry (data not shown). For the single-cell data, n = 48 (see also Figure S2C). (B) The mean number of replication forks during S phase (min. post-*cdc7-1*) inferred from the time-course data (gray lines represent the range covering 95% of the population). (C) Distance between active replication origins inferred from time-course data (continuous line; probability distribution) and from published DNA-combing data (histogram; Tuduri et al., 2010).

Single-cell measurements of locus replication times were used to validate the stochastic origin activation times derived from whole-genome replication-time-course data. The replication time of specific loci were assessed using fluorescence microscopy (Kitamura et al., 2006; Saner et al., 2013). Origin-proximal loci (*ARS727* and *ARS731*) were marked by *tet* and *lac* operator arrays, and the binding of fluorescent proteins allowed replication to be assayed by the increase in fluorescent dot intensity. Loci could either be replicated from the proximal origin (active replication) or by forks emanating from a more distant origin (passive replication). Note that *ARS726* is just 6 kb from *ARS727* and may have contributed to the early replication time of the nearby *tet* operator array. In each cell, both origin loci were marked and their replication time in multiple individual cells was determined (Figure 2A). From these measurements, we observed that each locus has a variety of replication times, thus providing direct evidence for stochastic origin activation time. Comparison of these single-cell measurements with equivalent values derived from the whole-genome time-course experiment revealed strikingly similar levels of stochasticity. These single-cell experiments confirmed the degree of stochastic origin replication time at several loci, providing support for the genome-wide values obtained from the ensemble time-course experiment.

Stochastic origin activation time gives rise to significant cell-to-cell variability in the pattern of genome replication. The data from the ensemble time-course experiment allowed the quantification of this variability and its comparison to independent experimental measures. For example, the number of active replication forks varies throughout S phase and across a population due to cell-to-cell differences in origin usage (Figure 2B). These derived fork numbers are consistent with biological estimates for the number of replisomes based on protein abundance in yeast cells (Ghaemmaghami et al., 2003; Mantiero et al., 2011; Tanaka et al., 2011). Furthermore, the stochastic activity of origins can cause occasional large distances between active origins. The population distribution for the distances between active origins was calculated from the whole-genome time-course data. The resulting distribution shows that large interactive origin distances are very rare, and it closely mirrors independent experimental estimates from published single-molecule experiments (Tuduri et al., 2010; Figure 2C).

In summary, the stochasticity observed in the whole-genome replication timing data can be accounted for by stochastic origin activity. Here, we provide direct supporting evidence for stochastic origin activation time from single-cell measurements. Furthermore, this variability in origin activity gives rise to variability in the number of replication forks and a range of distances between active origins, both of which we estimate and find to be comparable to independent experimental measurements.

### Genome-wide Quantification of Origin Efficiency

**Figure 3:**
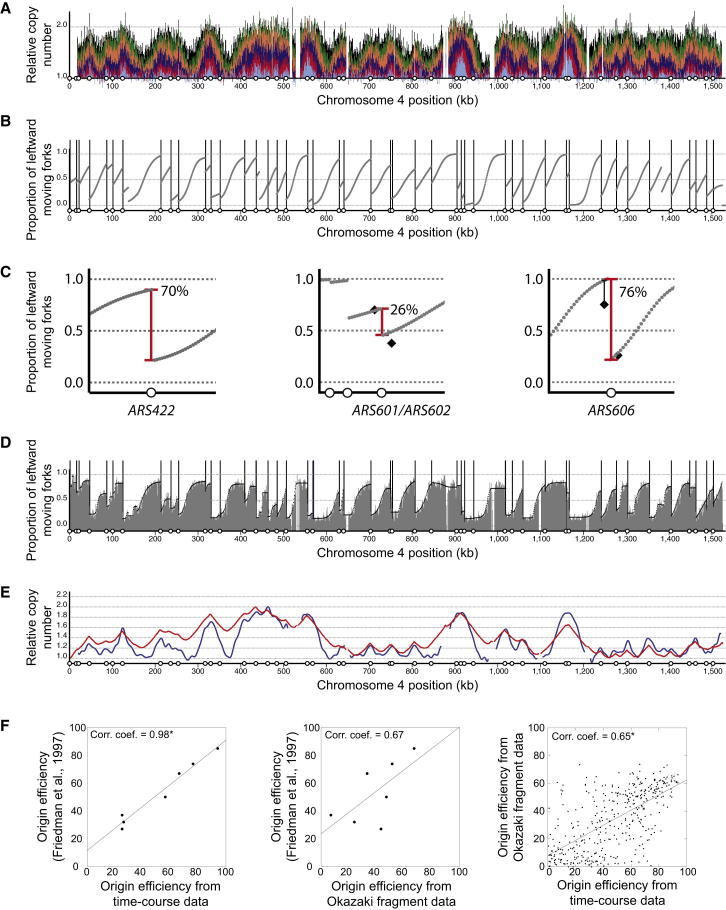
Genome-wide Replication Fork Direction and Origin Efficiency from Time-Course Data (A) Replication profile for chromosome 4 (as described for Figure 1A). (B) Proportion of leftward-moving forks across chromosome 4 inferred from replication-time-course data. Vertical lines mark the location of active origins, at each of which there is a sharp transition from leftward- to rightward-moving forks. (C) Proportion of leftward-moving forks for 50 kb regions centered on *ARS422* (left), *ARS601/ARS602* (center), and *ARS606* (right). Red vertical lines indicate the magnitude of the transition from leftward- to rightward-moving forks and the efficiency of the origin. Black diamonds indicate the proportion of leftward-moving forks at four chromosome 6 locations as previously determined by fork-direction gels (Friedman et al., 1997); for the location to the left of *ARS606*, the value was determined to be >75% and origin activity was estimated to be 74%. (D) Proportion of leftward-moving forks across chromosome 4 derived from mapping Okazaki fragments. Grey shading indicates the raw data with a fitted curve shown in black (origin locations are marked as in [B]; Smith and Whitehouse, 2012). (E) Replication time expressed as relative copy number derived from Okazaki fragment data (red; Smith and Whitehouse, 2012) or directly by deep sequencing (blue; Müller and Nieduszynski, 2012). (F) Pairwise comparisons of origin efficiency determined from the time-course data, from fork-direction gels (Friedman et al., 1997), and from mapping Okazaki fragments (Smith and Whitehouse, 2012). Pearson correlation coefficients are given (^∗^p < 0.0001).

One consequence of stochastic origin activity is that each cell uses a different cohort of origins in S phase. Interpretation of the replication-time-course data allowed the estimation of the efficiency of each origin in the genome. The gradient in replication time-course profiles (Figure 3A) corresponds to the proportion of forks that were moving left/rightward at each location in the genome (Baker et al., 2012; de Moura et al., 2010). Steep gradients indicate that a large proportion of forks were moving in one direction, whereas shallow gradients resulted from a mixture of left- and rightward-moving forks. Therefore, genome-wide fork direction can be determined from the replication profiles. In Figure 3B, the gray curve indicates the proportion of leftward-moving forks; regions above 0.5 (e.g., 845–900 kb) contained predominantly leftward-traveling forks, whereas regions below 0.5 resulted from a majority of forks moving rightward. Each of the data points contains analogous information to a fork-direction gel. Variations in the proportion of leftward-moving forks are a consequence of replication initiation or termination events. Active replication origins give rise to sharp changes in the predominant fork direction (Figure 3C). Therefore, these data allowed the determination of the efficiency of initiation and termination events. For example, upstream of origin *ARS422*, ∼90% of forks were traveling leftward, whereas downstream of the same origin, ∼20% were traveling leftward. Therefore, we can conclude that *ARS422* was active in ∼70% of cells (Figure 3C, left). The amplitude of these step sizes allows the determination of the efficiency of every genomic replication origin.

We compared inferred replication fork-direction data with direct experimental measurements. Fork-direction gels have been used to systematically determine the proportion of forks moving in each direction at a range of locations across chromosome 6 (Friedman et al., 1997). In Figure 3C, measurements from fork-direction gels are shown by black diamonds for locations flanking two example origins (*ARS601/ARS602* and *ARS606*). In each case, despite the difference in experimental approach, remarkably similar values for fork direction were obtained. Consequently, these independent approaches give near identical estimates of origin efficiency (see below).

A recent genome-wide study measured the density of Okazaki fragments (Smith and Whitehouse, 2012). These data provide a further independent experimental measure of fork direction (Figure 3D). In these experiments, Okazaki fragments were purified from cells undergoing a perturbed S phase (checkpoint-inactivated strain with a period of DNA ligase depletion). To test whether these perturbations altered global replication dynamics, we compared it with a replication-timing experiment that used an unperturbed S phase and was performed in the same strain background (Müller and Nieduszynski, 2012). Just as the gradients of replication profiles indicate the proportion of forks moving in each direction, the reciprocal mathematical transformation allowed relative replication time to be derived from fork-direction data (Baker et al., 2012; Retkute et al., 2012). In this way, relative replication time was directly compared between an Okazaki fragment purification experiment and a sort-seq experiment (Figure 3E). The positive correlation (correlation coefficient of 0.83) between the two measurements of relative replication time provides clear evidence that the perturbations employed to isolate Okazaki fragments do not dramatically alter the kinetics of DNA replication. Therefore, the whole-genome Okazaki fragment data provide a reliable measure of replication fork direction from which the efficiency of each origin can be estimated.

We compared the three independent experimental measures of replication origin efficiency: calculated from fork-direction gels (Friedman et al., 1997); inferred from the replication time-course experiment; and inferred from the mapping of Okazaki fragments (Smith and Whitehouse, 2012). Clear and statistically significant positive correlations were observed for the comparisons between origin efficiencies inferred from the time-course data and each of the other experimental measures (Figure 3F). The lack of a statistically significant correlation between fork-direction gels and the Okazaki fragment data may be a consequence of differences in the strain background (A364a and S288c, respectively), experimental noise, and/or the low number of data points. Comparison of the two independent genome-wide estimates of origin efficiencies revealed a notable number of outliers. These outliers might be explained by the strain differences or the difficulty in determining the efficiency of two closely spaced origins. There are examples where one approach called one member of an origin pair active and the other inactive and vice versa. Despite these caveats, there is a clear positive correlation between the independent approaches to determining origin efficiency, demonstrating that it is possible to quantify the variability in origin usage resulting from stochastic origin activity.

### Origin Activity Determines Termination Locations

**Figure 4:**
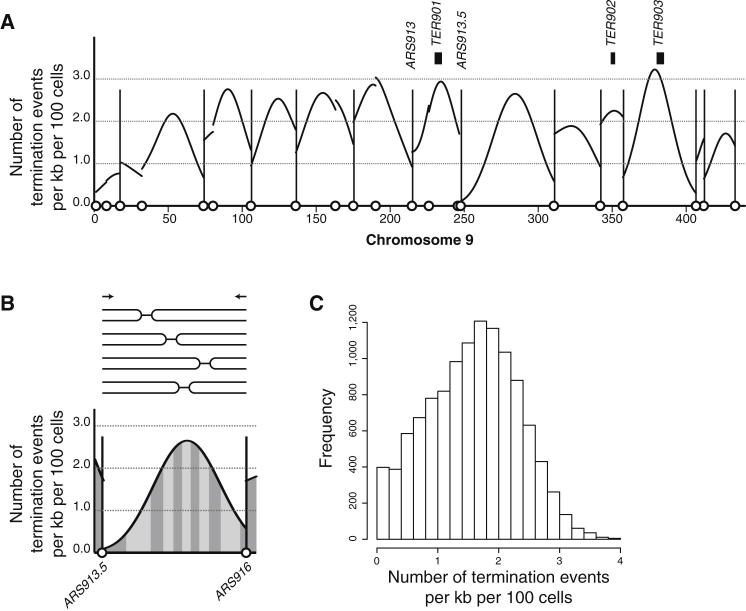
Replication Termination Events Are Dispersed Across the Genome (A) The distribution of replication termination events across chromosome 9 inferred from replication-time-course data. Replication origins are marked as in Figure 3B with two origins discussed in the main text labeled; previously described termination sites are marked (Fachinetti et al., 2010). Genome-wide data are shown in Figure S3. (B) Termination events between *ARS913.5* and *ARS916* are shown using exemplars above the inferred distribution. Alternately shaded areas each have an equal probability of a termination event (one termination event in every ten cells). (C) The genome-wide frequency distribution for the probability of termination events per kb per 100 cells.

We inferred the distribution of replication termination events from the replication-time-course data (Figures 4A and S3). The curve in Figure 4A shows the number of termination events per kb per 100 cells across chromosome 9. Termination events are widely distributed rather than located at a small number of isolated high-probability termination sites. Regions between active origins (marked with vertical lines in Figure 4A) contain a greater probability of termination events, but these are also spread over large ranges. A previous study mapped 71 termination sites (TERs) that together cover ∼3% of the genome (Fachinetti et al., 2010). These TERs coincide with loci identified as having a greater than average probability of a termination event (Figure 4A). However, ∼182 termination events (excluding telomeric events) were estimated per cell from the time-course data, of which only ∼8.7 (∼4.8% of the total) are within the TER sites. Therefore, these data are consistent with previously characterized TER sites, but the TER sites represent only a minority of all termination events.

Two stochastic components of replication origin activity give rise to a range of termination sites. First, variable origin efficiency results in different cohorts of origins being used between cells. Second, the relative activation time of two neighboring origins varies (Figure 2A), giving rise to a range of termination sites between active origins. For example, the active origins *ARS913.5* and *ARS916* (active in 74% and 56% of cells, respectively) gave rise to a dispersed distribution of replication termination events between them. The probability of a termination event is equal within each of the alternately shaded areas shown in Figure 4B. Across the whole genome, there is a similar pattern with replication termination events at virtually every location (Figure 4C). This dispersed distribution is consistent with replication termination sites being a consequence of stochastic origin activity, rather than sequences that favor termination.

**Figure 5:**
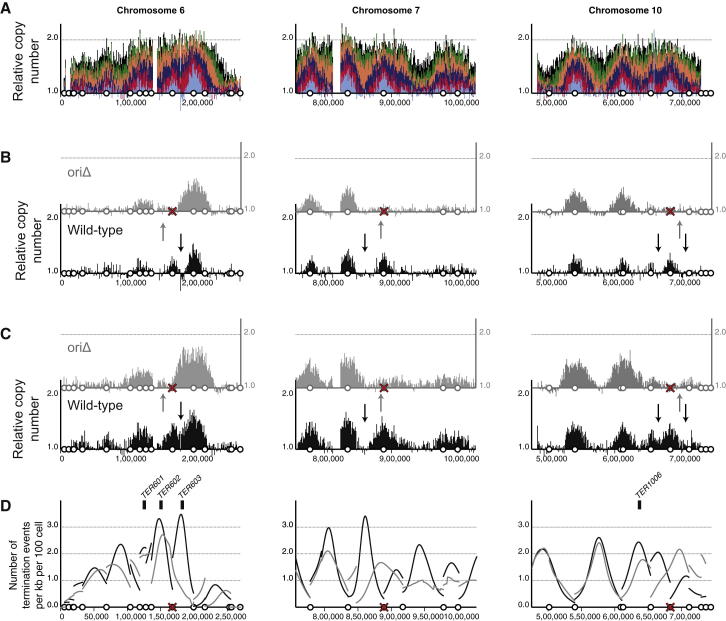
Inactivation of Replication Origins Results in Redistribution of Termination Events (A) Replication profile for chromosome 6 and regions of chromosomes 7 and 10 (as described for Figure 1A). (B and C) Comparisons of equivalent time points from the wild-type (black data) and origin mutant (gray data) are shown for each chromosomal region. Time points were selected to ensure equivalent levels of genome-wide DNA synthesis; time points are 15 and 20 min (wild-type) and 20 and 25 min (origin mutant) in (B) and (C), respectively. Vertical arrows indicate the inferred location of the highest probability termination sites. (D) The inferred distribution of replication termination sites for the wild-type (black) and the origin mutant (gray). Data displayed as in Figure 4A. A red cross marks origins that were inactivated in the mutant strain (see also Figures S4 and S5).

To test whether replication origin activity is responsible for the location of termination events, we inactivated three active origins: *ARS606*, *ARS731.5*, and *ARS1021* (Figure 5A). At each of these origins, origin recognition complex (ORC) binding was abolished by introducing a four-base-pair mutation in the ORC-binding site at the native chromosomal loci. The introduced mutations eliminate origin activity on a plasmid (Figure S4). The replication dynamics of this triple-origin mutant were characterized as described above for the wild-type. The profiles show that unperturbed chromosomes replicate similarly to the wild-type (Figure S5). Regions of chromosomes 6, 7, and 10, that are distant from the inactivated origins, also replicate with similar kinetics to the wild-type (Figures 5B and 5C). Comparison of early S phase time points from the wild-type and origin mutant revealed the lack of copy number peaks associated with the mutated origins (Figures 5B and 5C). Therefore, the ORC-binding site mutations abolished chromosomal origin function.

Equivalent time points from the wild-type and origin mutant experiments were compared to determine the consequences for location of replication termination events. Between *ARS606* and *ARS607*, there is a region with an elevated probability of replication termination events (Figure 5D, left); this region overlaps with the previously described *TER603* (Fachinetti et al., 2010). The location with the greatest probability of a replication termination event is at 180,500 bp (black arrows in Figures 5B and 5C). This coincides with the location of a tRNA gene, a feature that has previously been implicated in replication termination. However, in the absence of *ARS606* activity, there is a dramatic reduction in probability of replication termination events at this location (Figure 5D, left). Furthermore, there is no evidence for a delay in replication fork progression through this region (gray data in Figures 5B and 5C). Inactivation of *ARS731.5* and *ARS1021* alter the distribution of termination events in an analogous manner. In each case, when origins are inactivated, the most probable location for termination events clearly moves (Figure 5D). We conclude that particular genomic features do not fix the location of termination events; instead, they are determined by the activity of the flanking origins.

## Discussion

Here, we show that deep sequencing measurements of copy number changes during a synchronous S phase produce the spatial and temporal resolution required to perform a highly quantitative analysis of genome replication. Combining experimental data with a mathematical model (Retkute et al., 2011) enabled the estimation of the behavior of individual origins, including the stochastic properties. This demonstrates that the stochastic characteristics of individual genomic loci can be inferred from high-resolution ensemble data.

The mathematical model used to interpret the data made the assumption that replication fork velocity is on average constant, an assumption with wide experimental support (de Moura et al., 2010; Sekedat et al., 2010; Tuduri et al., 2010; Yang et al., 2010). This model allowed the inference of the stochastic properties of genome replication, including variation in origin activation time and competence, from high-resolution population ensemble time-course data. These stochastic properties leave a signature in the ensemble data that is not altered by the degree of cell-cycle synchronization within the population (de Moura et al., 2010; Retkute et al., 2011). Single-cell and single-molecule measurements were consistent with the estimated variability in origin activity (Figure 2). As reported previously (Yang et al., 2010), there is greater variability in the activation time of later-activating origins. This is consistent with origin activation time being a stochastic process, for example determined by affinity for a limited number of activating molecules (Douglas and Diffley, 2012). Crucially, stochastic origin activity is responsible for variability in genome replication, including differences in origin usage and sites of replication termination. As discussed below, these differences can contribute to genome stability, thus underlining the importance of stochastic origin activity.

We have estimated the proportion of S phases in which each replication origin activates, referred to as the efficiency of an origin. There is a strong correlation between these estimates and those previously determined for origins on chromosome 6 (Figures 3C and 3F). Across the genome, 459 origin sites (Siow et al., 2012) were considered, of which an estimated average of ∼285 sites are licensed and ∼198 activated per cell (Table S1). Those licensed sites that do not activate (dormant origins) are passively replicated and are potential backup origins, available to rescue replication if forks stall irreversibly (Blow et al., 2011; Newman et al., 2013). Therefore, the stochastic nature of origin activation time gives rise to dormant origins that can contribute to surviving replicative stress and ensuring genome stability.

Replication initiation sites are discrete chromosomal loci defined by the binding of the ORC and the Mcm2-7 complex. By contrast, replication termination events are widely distributed (Figure 4). In a population of cells, the majority of the genome (>75%; Figure 4C) is within 1 kb of a termination event in at least 1% of cells. For context, the most probable termination sites will only experience a termination event per kb in 3.9% of cells. The distribution of termination sites that we find is consistent with and greatly extends previously reported termination (TER) sites (Fachinetti et al., 2010). Those TER sites were found to colocate with particular chromosomal features, including tRNAs and centromeres. However, our observation that these sites constitute only a small minority of all termination events, combined with observations that such sites do not pause replication forks (Azvolinsky et al., 2009), suggest that they do not directly influence termination, although we note that the current available resolution does not rule out the possibility that particular sequence properties might influence the precise location of termination events. Perturbing genome replication, by the inactivation of three active replication origins, tested how termination sites are specified. The inactivated origins did not globally alter the pattern of genome replication, allowing investigation of the consequences for termination. The inactivations resulted in changes in the location of termination sites, consistent with origin activity being the principal influence over the distribution. Recently reported genome-wide perturbations to DNA replication origin activity also resulted in relocalization of termination events (McGuffee et al., 2013). These findings are consistent with the observed distribution of base substitutions in a mutator strain, which were hypothesized to be a consequence of considerable variability in fork termination sites (Larrea et al., 2010). We conclude that the diversity in termination sites can be explained by the variability in origin usage and stochastic activation time—both likely to be properties of origin function in all eukaryotes.

Therefore, replication termination in eukaryotes contrasts with the highly regulated termination (with replication fork traps) in bacteria (Duggin et al., 2008). A consequence of the diversity in termination sites (and variability in origin usage) observed in eukaryotes is that there will be variability in which regions of the genome are latest replicating between cells within a population. Both replication termination and late replication per se have been linked to genome instability. Late replication is associated with fragile sites (Letessier et al., 2011) and elevated mutation rates (Agier and Fischer, 2012; Lang and Murray, 2011; Stamatoyannopoulos et al., 2009; Weber et al., 2012). Failure to correctly resolve merging replication forks during termination can result in unreplicated DNA and pathology (Rudolph et al., 2013; Steinacher et al., 2012). Although eukaryotes have multiple mechanisms to limit these errors, a small proportion will escape repair and contribute to genome instability. By dispersing these sites across the genome, eukaryotes may limit the potentially deleterious accumulation of mutations at particular loci.

## Experimental Procedures

### Yeast Strains and Cell-Cycle Synchrony

The wild-type strain was RM14-3A (*MATa cdc7-1 bar1 his6 trpl-289 ura3-52 leu2- 3,7 12*, A364a background [McCarroll and Fangman, 1988]). The triple autonomously replicating sequence consensus sequence (ACS) mutant strain was an RM14-3A derivative constructed by sequentially replacing the ACS at *ARS606*, *ARS731.5*, and *ARS1021* with an inactivated version (4 bp mutation). The resulting strain was backcrossed three times to produce the strain (MHY200) used for deep sequencing. To visualize loci near *ARS727* and *ARS731*, tetOx224 (11.2 kb) and lacOx256 (10.1 kb) were integrated by a two-step “pop-in and pop-out” method. tetOx224 was integrated to chromosome 7 at 660,847 bp (from the left telomere) within the *ARS727* replicon. lacOx256 was integrated to chromosome 7 at 842,709 bp within the *ARS731* replicon (Saner et al., 2013). Plasmids and oligonucleotide sequences are available upon request.

Cells grown in standard rich YPD media were treated with 200 nM α factor for 4 hr at 23°C. Cells were then shifted to 38°C and treated with 0.2 mg/ml pronase. After 2.5 hr at 38°C, cells were cooled rapidly to 23°C. The cell culture was incubated at 23°C and samples collected every 2.5 min for flow cytometry analysis and every 5 min for isolation of genomic DNA.

### Deep Sequencing

Deep sequencing was performed on the AB SOLiD 4 analyzer platform. Sequencing libraries were made using the NEB Next kit (New England Biolabs) as advised by the manufacturer. Each sequencing sample was assigned 1/16 of an AB SOLiD sequencing slide. Resulting reads were mapped to the 2003 *S. cerevisiae* reference genome using Bioscope 1.3.1 (Life Technologies). Each sequenced sample yielded 10–25 million 50 bp reads, equivalent to ∼50- to 100-fold coverage per base.

### Data Analysis

Replication timing profiles were generated by calculating the ratio of uniquely mapping reads in the replicating (S phase) samples to the nonreplicating (*cdc7-1* arrest) sample (Müller et al., 2013). Custom Perl scripts were used to independently calculate this ratio for every 1 kb window. Windows where fewer than 250 reads were mapped in either sample were excluded. Differences in absolute read number were normalized to give an average copy number ratio of one. The modal bulk DNA content at each time point was measured using flow cytometry, and a curve was fitted. Values from this curve were used for normalization, so that each time point had a mean relative copy number equivalent to the proportion of the genome replicated at that time point.

### Mathematical Modeling

An existing DNA replication model (de Moura et al., 2010; Retkute et al., 2011, 2012) was extended to the whole genome. Origin activation time distributions were described by a Hill’s type function with median activation time T_1/2_ and the width of the activation distribution T_w_. Model equations and derived quantities are given in the Supplemental Experimental Procedures.

A genetic algorithm was used to estimate fork velocities and origin parameters (p, T_1/2_, and T_w_) by minimizing the sum of the square of the differences between experimentally measured relative copy number data and model estimations. An existing open-source implementation was used (http://ftp.mcs.anl.gov/pub/pgapack/).

### Fluorescence Microscopy

The procedures for live-cell imaging were described previously (Kitamura et al., 2006). Briefly, time-lapse images were collected at 25°C (ambient temperature). For image acquisition, we used a DeltaVision RT microscope (Applied Precision), UPlanSApo 100× objective lens (Olympus; NA 1.40), a CoolSnap HQ CCD camera (Photometrics), and SoftWoRx software (Applied Precision). Cyan fluorescent protein and GFP signals were discriminated with the 89006 ET filter set (Chroma). We acquired nine z-sections (0.7 μm apart), which were subsequently analyzed with Volocity (Improvision) software. Replication timing of the loci, where tetO and lacO arrays were integrated, was determined as described previously (Kitamura et al., 2006; Saner et al., 2013).

## Accession Numbers

The NCBI Gene Expression Omnibus (http://www.ncbi.nlm.nih.gov/geo/) accession number for the data reported in this paper is GSE48561.

## Supplemental Information

Document S1. Extended Experimental Procedures and Figures S1–S5

Table S1. Replication Origin Properties Inferred from the Replication-Time-Course data Each line corresponds to a replication origin and gives the following information: the chromosome (A), position (B), inferred median activation time (C), inferred width of the activation distribution (D), competence (E) and efficiency (F). In addition, the efficiency of each origin as inferred from the Okazaki fragment data (Smith and Whitehouse, 2012) is given (G).

Document S2. Article plus Supplemental Information
