# Supplementary material for: High-Resolution Replication Profiles Define the Stochastic Nature of Genome Replication Initiation and Termination
Source: Cell Rep. 2013 Nov 7;5(4):1132–41. doi: 10.1016/j.celrep.2013.10.014 (PMC3898788; doi:10.1016/j.celrep.2013.10.014)
Supplement: Document S1. Extended Experimental Procedures and Figures S1–S5 [file mmc1.pdf]

Cell Reports, Volume *5*
Supplemental Information

High-Resolution Replication Profiles Define the Stochastic Nature of Genome Replication Initiation and Termination

Michelle Hawkins, Renata Retkute, Carolin A. Müller, Nazan Saner, Tomoyuki U. Tanaka, Alessandro P.S. de Moura, and Conrad A. Nieduszynski

Due to the restrictions on supplemental figures, genome-wide data plots for the various datasets shown with exemplar chromosomes within manuscript figures will be made available for download from the author's website.

Supplemental Material

Extended Experimental Procedures

Mathematical model

The DNA replication model (de Moura et al., 2010; Retkute et al., 2011, 2012) has been extended to the whole genome.

The genome has K chromosomes, and we assume that each chromosome is replicated independently. Each chromosome has n_j origins, length N_i bp, and forks move with constant velocity of v_i, $i=1..K$.

Each origin j on chromosome i is described by position x_{ij}, competence p_{ij}, average activation time $T_{1/2}^{ij}$ and the width of the activation distribution T_w^{ij}. The origin activation functions is $q_{ij}(t) \equiv p_{ij} f\left(t, T_{1/2}^{ij}, T_w^{ij}\right)$, with the shape of activation distribution described by Hill's-type function:

$$f\left(t, t_{1/2}, t_w\right) = \frac{\ln(3) t_{1/2} \left(t_{1/2}/t\right)^{\ln(3)/\kappa - 1}}{\kappa t^2 \left(1 + \left(t_{1/2}/t\right)^{\ln(3)/\kappa}\right)^2},$$

where

$$\kappa = \ln\left(\frac{t_w + \sqrt{4t_{1/2}^2 + t_w^2}}{2t_{1/2}}\right).$$

Figure 1F shows the probability of activation for origins *ARS606* and *ARS1412*.

The probability density of an origin being activated t minutes after *cdc7-1* release is given by following equation:

$$q_{ij}^{ACT}(t) = \frac{1}{1 - \prod_{k=1}^{n_i} \left(1 - p_{ik}\right)} q_{ij}(t) \prod_{k \neq i} \left(1 - p_{ik} + \int_t^{\infty} q_{ik}\left(\tau - |x - x_{ik}|/v_i\right) d\tau\right).$$

The probability density of an origin being passively replicated t minutes after *cdc7-1* release is:

$$q_{ij}^{PAS}(t) = \frac{1}{1 - \prod_{k=1}^{n_i} \left(1 - p_{ik}\right)} \sum_{k=1}^{j-1} q_{ik}(t) \prod_{l \neq k} \left(1 - c_{il} + \int_t^{\infty} q_{il}\left(\tau - |x - x_{il}|/v_i\right) d\tau\right)$$
$$+ \frac{1}{1 - \prod_{k=1}^{n_i} \left(1 - p_{ik}\right)} \sum_{k=j+1}^{ni} q_{ik}(t) \prod_{l \neq k} \left(1 - c_{il} + \int_t^{\infty} q_{il}\left(\tau - |x - x_{il}|/v_i\right) d\tau\right).$$

Then the probability of loci replication with respect to time is given by the sum of the probability density of the origin being activated and the probability

density of the origin being passively replicated at t minutes after *cdc7-1* release, i.e. $q_{ij}^{ACT}(t)+q_{ij}^{PAS}(t)$. Figure 2A shows the probability of replication with respect to time for origins *ARS727* and *ARS731*.

The relative copy number for position x on chromosome j at time t is:

$$CN_i(x,t)=\frac{1}{1-\prod_{j=1}^{n_i}(1-p_{ij})}\int_{-\infty}^{t}\sum_{j=1}^{n_i}q_{ij}\left(t-|x-x_{ij}|/v_i\right)\prod_{k\neq j}\left(1-p_{ik}+\int_t^{\infty}q_{ik}\left(\tau-|x-x_{ik}|/v_i\right)d\tau\right)d\tau$$

Model parameters were estimated by fitting the above equation to the experimental data. Figure 1H shows a fitted model for chromosome 14 at 15 min, 25 min and 35 min after *cdc7-1* release.

The average number of replication forks in a single cell is given by:

$$n_f(t)=\sum_{i=1}^{K}\frac{1}{v_i\left(1-\prod_{j=1}^{ni}(1-p_{ij})\right)}\int_0^{N_j}\sum_{j=1}^{n_i}q_{ij}\left(t-|x-x_{ij}|/v_i\right)\prod_{k\neq j}\left(1-p_{ik}+\int_t^{\infty}q_{ik}\left(\tau-|x-x_{ik}|/v_i\right)d\tau\right)dx$$

Figure 2B shows average number of replication forks (black solid line) together with 5th and 95th percentiles (grey solid line).

The average number of origins activated in a single cell is given by:

$$n_O(t)=\sum_{i=1}^{K}\sum_{j=1}^{n_i}\frac{1}{1-\prod_{k=1}^{n_i}(1-p_{ik})}\int_{-\infty}^{\infty}q_{ij}(t)\prod_{k\neq j}\left(1-p_{ik}+\int_t^{\infty}q_{ik}\left(\tau-|x-x_{ik}|/v_i\right)d\tau\right)dt.$$

Median replication time for position x on chromosome i is:

$$T_i(x)=\frac{1}{1-\prod_{j}^{n_i}(1-p_{ij})}\int_{-\infty}^{\infty}t\sum_{j=1}^{n_i}q_{ij}\left(t-|x-x_{ij}|/v_i\right)\prod_{k\neq j}\left(1-p_{ik}+\int_t^{\infty}q_{ik}\left(\tau-|x-x_{ik}|/v_i\right)d\tau\right)dt.$$

Proportion of leftward moving forks for position x on chromosome i is:

$$LF_i(x)=\left(1-v_iT_i'(x)\right)/2.$$

Rearranging and integrating the above gives the expression to calculate the median replication time from the proportion of leftward moving forks:

$$T_i(x)=\frac{1}{v_i}\int\left(1-2LF_i(x)\right)dt\,.$$

Probability of termination events for position x on chromosome j and at time t is:

$$P_i^{term}(x)=\frac{1}{A}\int_{-\infty}^{\infty}\sum_{j=1}^{n_i}\sum_{k=j+1}^{n_i-1}I\left(x_j<x<x_k\right)q_{ij}\left(t-\left|x-x_j\right|/v_i\right)q_{ik}\left(t-\left|x-x_k\right|/v_i\right)$$
$$\times\prod_{l\neq j,l\neq k}\left(1-p_{il}+\int_t^{\infty}q_{jl}\left(\tau-\left|x-x_l\right|/v_i\right)d\tau\right)dt$$

where A is a normalisation constant and indicator function *I(g)=1* if *g* is true. We have normalised the probability such that the area under the curve over each chromosome is equal to a number of termination event, i.e. $\sum_{j=1}^{n_i}p_{ij}-1$.

The probability density function for inter-active-origin distances is:

$$P^{IOD}(y)=\frac{1}{A}\int_{-\infty}^{\infty}\sum_{i=1}^{K}\sum_{j=1}^{n_i}\sum_{k=j+1}^{n_i-1}I\left(y=\left|x_j-x_k\right|\right)q_{ij}(t)q_{ik}(t)\prod_{l=1}^{j-1}\left(1-p_{il}+\int_t^{\infty}q_{il}\left(\tau-\left|x_j-x_l\right|/v_i\right)d\tau\right)$$
$$\times\prod_{l=k+1}^{n_i}\left(1-p_{il}+\int_t^{\infty}q_{il}\left(\tau-\left|x_k-x_l\right|/v_i\right)d\tau\right)\prod_{l=j+1}^{k-1}\left(1-p_{il}+\int_t^{\infty}q_{il}(\tau)d\tau\right)dt$$

Figure 2C shows a model estimated probability density function for inter-active-origin distances (solid line).

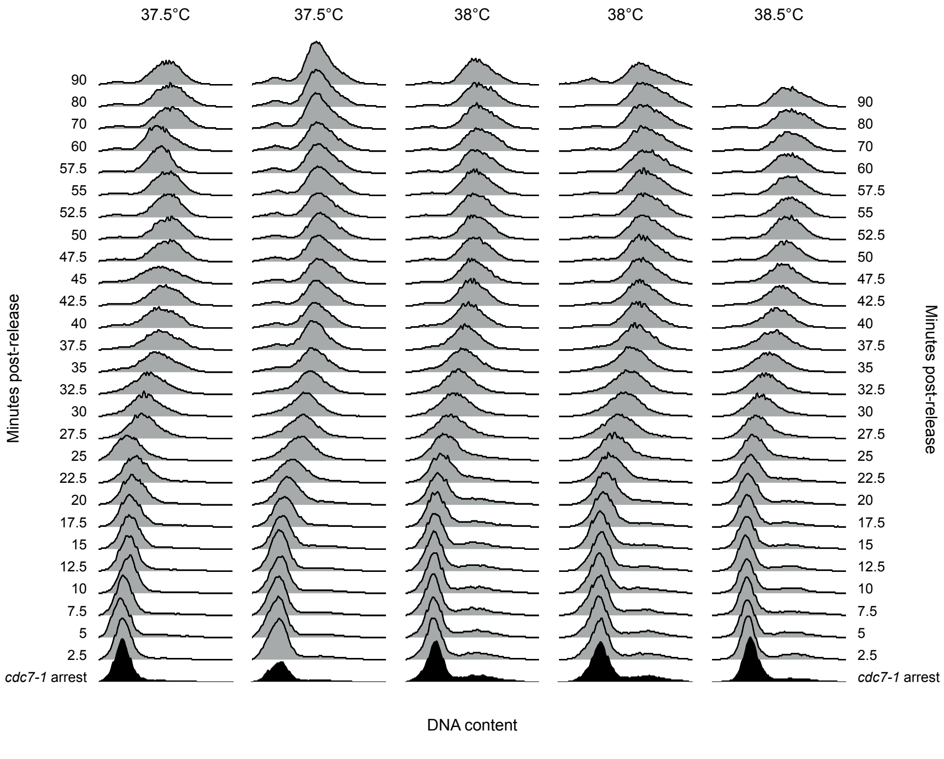

Figure S1: Optimization of the *cdc7-1* arrest temperature, Related to Figure 1. Flow cytometry data for a series of cell cycle experiments. Each culture was arrested first in alpha factor and then released with pronase at the indicated temperatures to arrest prior to DNA replication. Cultures were released from the *cdc7-1* arrest by rapid cooling to 23 °C and samples for flow cytometry were taken every 2.5 minutes.

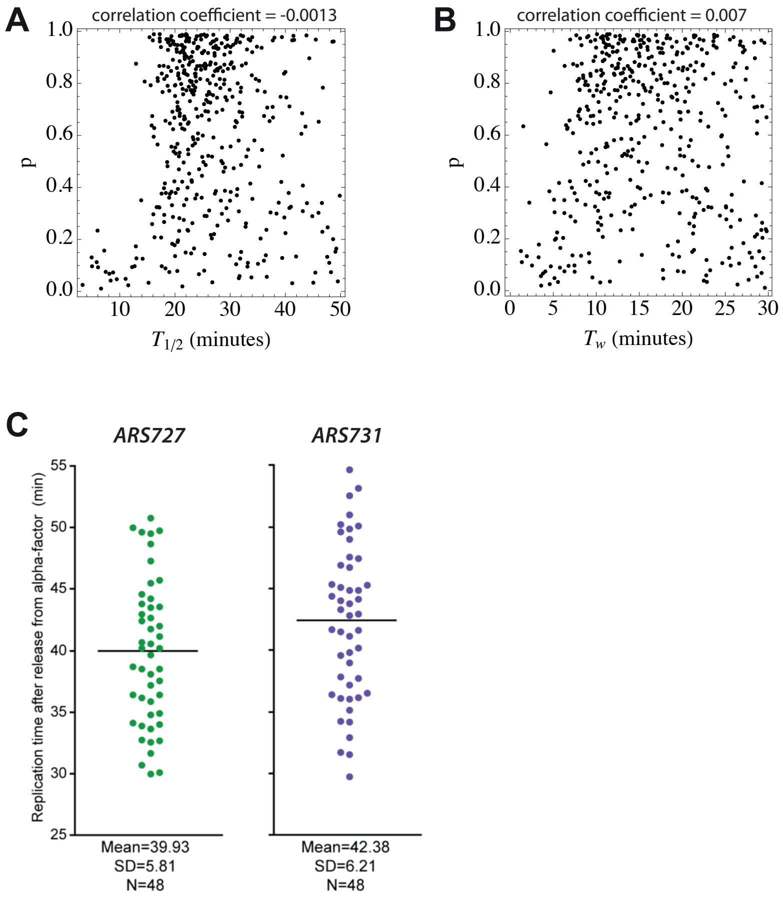

Figure S2: Comparison of replication origin parameters and single cell replication timing data, Related to Figure 1 & 2. (A & B) No correlation was observed between origin competence and activation time. (C) Replication timing data for 48 single cells for the *tet* array proximal to *ARS727* (left) and the *lac* array proximal to *ARS731* (right).

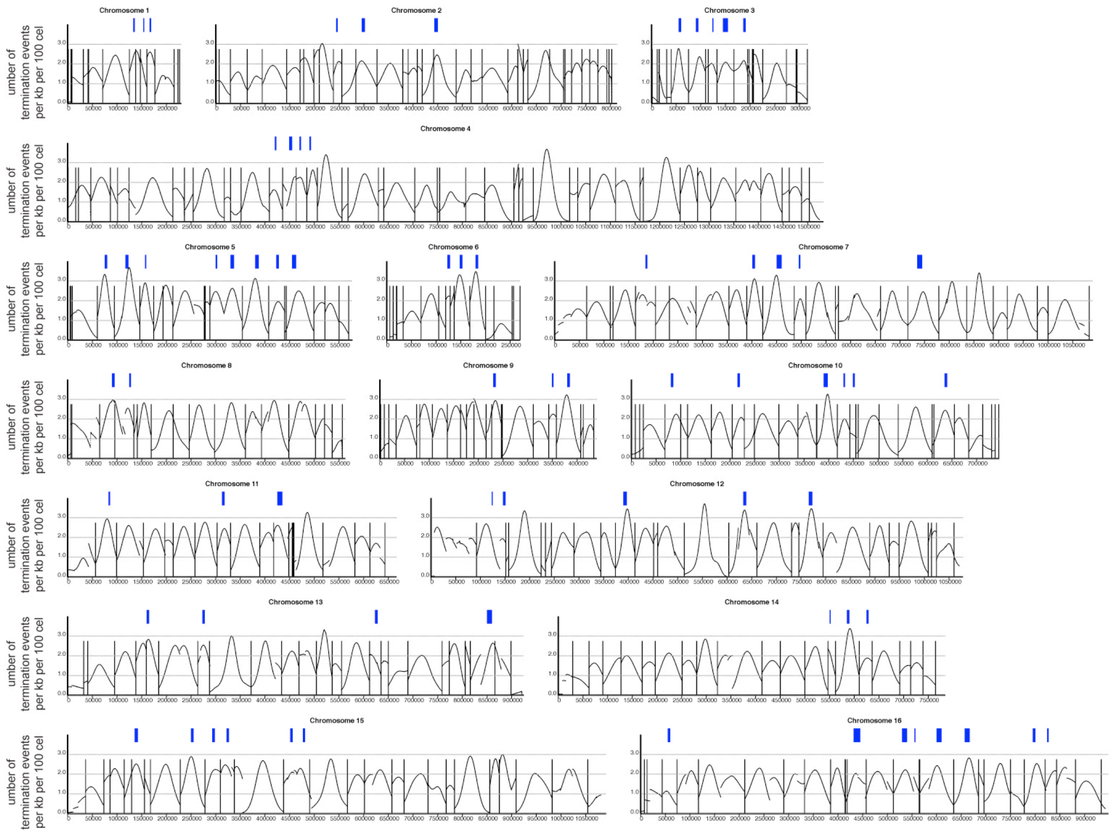

Figure S3: Genome-wide distribution of replication termination events, Related to Figure 4. The distribution of replication termination events inferred from replication time-course data. Replication origins are marked by black vertical bars; previously described termination sites are marked in blue above each chromosome (Fachinetti et al., 2010).

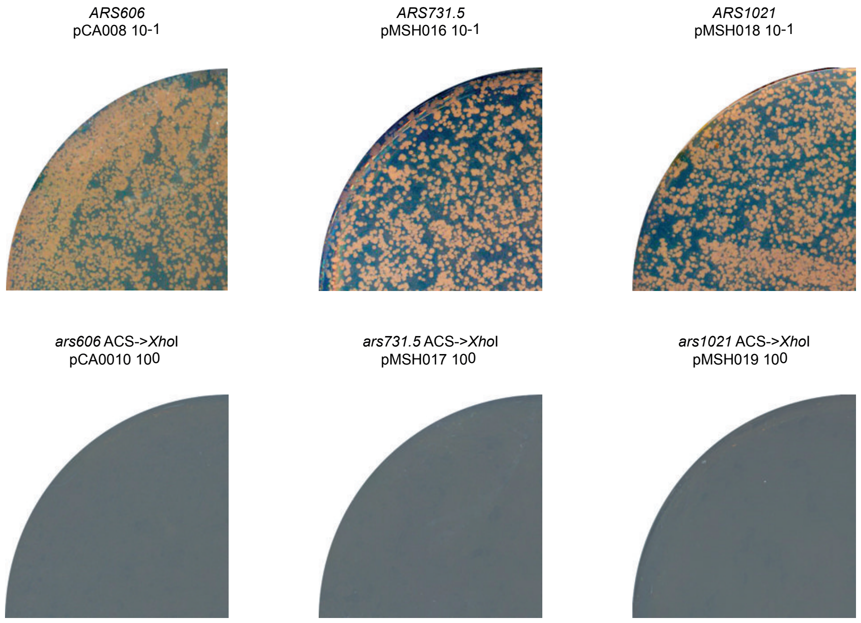

Figure S4: Mutational inactivation of replication origins, Related to Figure 5. Plasmid-based assays to demonstrate that the introduced mutations inactivate *ARS606*, *ARS731.5* and *ARS1021*.

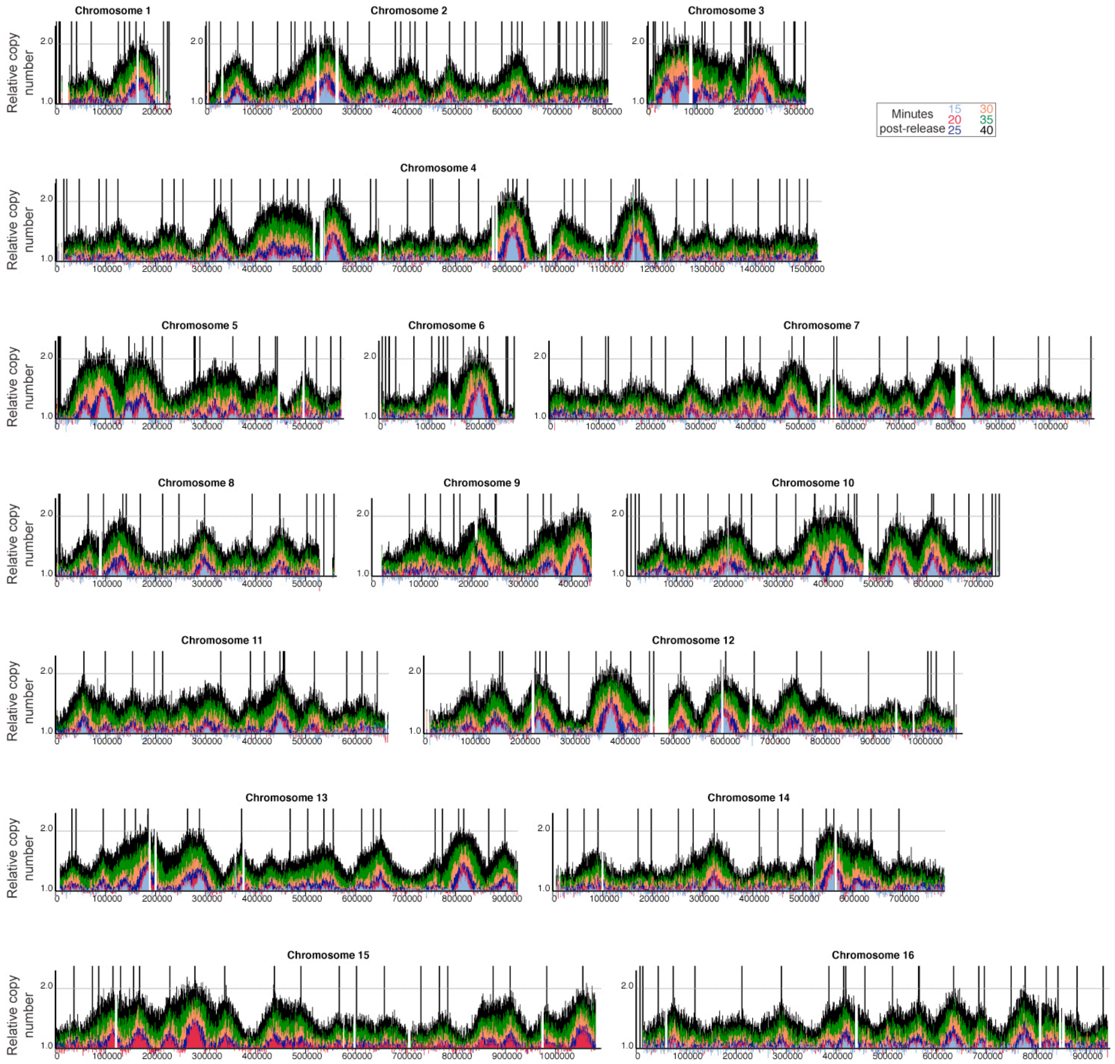

Figure S5: Genome-wide replication profiles for the triple origin mutant strain, Related to Figure 5. The six S-phase time-points (15, 20, 25, 30, 35 and 40 min) are relative to release from the *cdc7-1* zero time-point. Each data point represents the extent of DNA replication in a 1kb window. Black vertical bars represent the location of replication origins.

Supplemental table legend

Table S1: Replication origin properties inferred from the replication time-course data. Each line corresponds to a replication origin and gives the following information: the chromosome (column A), position (B), inferred median activation time (C), inferred width of the activation distribution (D), competence (E) and efficiency (F). In addition the efficiency of each origin as inferred from the Okazaki fragment data (Smith and Whitehouse, 2012) is given (column G).

Supplemental references

de Moura, A.P., Retkute, R., Hawkins, M., and Nieduszynski, C.A. (2010). Mathematical modelling of whole chromosome replication. Nucleic Acids Res *38*, 5623-5633.

Fachinetti, D., Bermejo, R., Cocito, A., Minardi, S., Katou, Y., Kanoh, Y., Shirahige, K., Azvolinsky, A., Zakian, V.A., and Foiani, M. (2010). Replication termination at eukaryotic chromosomes is mediated by Top2 and occurs at genomic loci containing pausing elements. Molecular Cell *39*, 595-605.

Retkute, R., Nieduszynski, C.A., and de Moura, A. (2011). Dynamics of DNA replication in yeast. Phys Rev Lett *107*, 068103.

Retkute, R., Nieduszynski, C.A., and de Moura, A. (2012). Mathematical modeling of genome replication. Phys Rev E *86*, 031916.

Smith, D.J., and Whitehouse, I. (2012). Intrinsic coupling of lagging-strand synthesis to chromatin assembly. Nature *483*, 434-438.
